# Investigating the Relationship between Alcohol Consumption and the Development of Alzheimer's Disease

**DOI:** 10.1002/alz70856_107344

**Published:** 2026-01-09

**Authors:** Carly Mitchell, Dorea P Jenkins, Brittany T Ivey, Jordan Logue, Erik Wilkes, Shelby Carter, Judson L Chandler, Lori L McMahon, Steven L Carroll

**Affiliations:** ^1^ Medical University of South Carolina, Charleston, SC, USA; ^2^ University of Virginia, Charlottesville, VA, USA

## Abstract

**Background:**

Alzheimer's disease (AD) in the United States is predicted to double by 2060 from 6.9 to13.8 million cases. AD is characterized by the abnormal buildup of beta‐amyloid plaques and irregular protein tau structures in various regions of the brain. This accumulation of protein impairs memory and cognitive function that disrupts the daily life of those afflicted with the disease. Factors for developing AD are age, genetics, health conditions and lifestyle choice, such as Alcohol Use Disorder. Alcohol Use Disorder can independently promote damage, accumulation of plaques, vascular injury and neuroinflammation to the areas of the brain. Early diagnosis of these changes is paramount to detection of AD and associated diseases, this has been shown to be possible by assessing the eye. We hypothesize that morphological changes associated with AD and other neurodegenerative markers (established and novel) in the ocular tissue of our rat model that will help to clarify the relationship between alcohol consumption and AD development.

**Method:**

Our collaborators exposed wild type and TgF344‐AD rats, an AD model heterozygous for human mutant APPswe/PS1dE9, ages 4‐7 months to 200 proof alcohol in vaporization chambers. Treatment was administered intermittently, acutely or chronically, during adolescence (≈ p28‐p56) and/or adulthood (≈ p70‐p98). Experimental groups included rats that 1) did not receive alcohol at all or received alcohol 2) in adolescence only, 3) in adulthood only, or 4) in both adolescence and adulthood. Rats also received air only as a control treatment. We obtained eyes from these rats and subjected them to histological processing and staining with hematoxylin and eosin (H&E) and several known and novel markers of neurodegeneration. Our board‐certified neuropathologist reviewed reported on each stain.

**Result:**

A total of 180 eyes were harvested from our experimental rat groups. Extensive β‐amyloid staining has been performed in brains harvested from these animals’ following treatment. Additionally, pTau staining with CP13 is currently being executed. We plan to correlate staining from brain tissue with ocular tissue.

**Conclusion:**

Identifying novel biomarkers associated with alcohol consumption can provide novel insights into how lifestyle choices contribute to AD development.

